# Effectiveness and safety of enzyme replacement therapy in the treatment of Fabry disease: a Chinese monocentric real-world study

**DOI:** 10.1186/s13023-024-03441-1

**Published:** 2024-11-11

**Authors:** Yingjie Liu, Ying Li, Pei Li, Songyun Zhang, Zhang Zhiqing

**Affiliations:** 1 The Second Hospital of Hebei Medical University, Shijiazhuang, 050000 China; 2 Hebei Key Laboratory of Rare Diseases, Shijiazhuang, 050000 China

**Keywords:** Fabry disease, Rare disease, Enzyme replacement therapy, Effectiveness, Safety, Real-world study

## Abstract

**Objective:**

To assess the effectiveness and safety of enzyme replacement therapy (ERT) for treating Fabry disease in clinical practice.

**Methods:**

The clinical data of patients with Fabry disease were retrospectively collected and screened according to inclusion and exclusion criteria. The effectiveness of ERT was evaluated by analyzing the improvement in renal dysfunction (decreased estimated glomerular filtration rate (eGFR) and proteinuria), cardiac system injury (mainly increased left ventricular mass index (LVMI)), and neuropathic pain after ERT treatment. The safety of ERT was measured by summarizing the occurrence of adverse events (AE) and adverse drug reactions (ADR) before and after ERT.

**Results:**

Sixteen patients with Fabry disease who underwent ERT treatment 2–36 times over a period of 2–89 weeks were enrolled in the study. Among them, 13 received symptomatic treatment based on the involvement of various organs, 14 were treated with anti-inflammatory and anti-allergic drugs, and 16 had no AE or ADR. After ERT, there was no significant difference in (eGFR, microalbumin (mALB), 24 h urinary protein quantitation (24 h PRO), urinary albumin/creatinine ratio (ACR), uric acid (UA), and β2 microglobulin (β2MG) (*P* > 0.05), and the renal function remained stable or improved; ERT could significantly reduce left ventricular mass index (LVMI) (*P* = 0.043) and lactate dehydrogenase (LDH) (*P* = 0.031), and other cardiac function indexes had an improvement trend or remained stable, but the difference was not significant (*P* > 0.05). After ERT, the degree of limb pain in three of the four minor patients improved.

**Conclusions:**

ERT could effectively stabilize or improve renal and cardiac function and relieve neuropathic pain in patients with Fabry disease, and no AE occurred during treatment, and the clinical effectiveness and safety were satisfactory.

## Introduction

Fabry disease is a rare hereditary disease with an incidence of 1/117000–1/50000 in the global population [1], 1/117000–1/10000 in the United States [2–4], and 1/15000 in Europe [5]. The incidence in certain populations is even higher. Newborn screening measures have found an unexpectedly high prevalence of the disease, ranging from 1/8800 to 1/1250 [6–8]. In China, the incidences are 0.12% in patients undergoing dialysis for end-stage renal failure dialysis patients [9] and 0.93–1.6% in patients with left ventricular hypertrophy (LVH) [10, 11]. Fabry disease is a typical X-linked genetic lysosomal storage disease [12], caused by a α-galactosidase A (GLA) gene mutation on chromosome Xq22, leading to a complete or partial loss of α-galactosidase A (α-Gal A) activity, blocking the degradation of substrate globotriaosylceramide (GL-3) and its derivative globotriaosylsphingosine (Lyso-GL-3), which are accumulated in various organs and tissues of the human body, resulting in multiple organ damage [13]. Severe complications, including renal dysfunction, cardiac and cerebrovascular events and neuropathic pain, emerge in severe cases [14], resulting in poor quality of life and a high risk of premature death, which reduce the life expectancy of male patients by 15–20 years and female patients by 6–10 years [15].

For treating the pathogenesis of Fabry disease, specific strategies include reducing substrate production and enhancing enzyme activity. Enzyme Replacement Therapy (ERT) is the regular infusion of gene recombinant enzymes to replace α-Gal A with reduced or lost activity in patients and degrade the stored substrate to relieve clinical symptoms [16, 17]. In 1973, Brady et al. [18] first attempted ERT in the treatment of Fabry disease and achieved satisfactory results. Since 2001, ERT has been gradually promoted and applied in clinical practice in China [9], the United States [19], the European Union [20, 21], and other countries, including ERT-specific treatment in the diagnosis and treatment guidelines of Fabry disease, which has significantly improved the clinical treatment effect. Two ERT drugs are currently available in clinical settings: agalsidase α (Replagal^®^, Takeda) and agalsidase β (Fabrazyme^®^, Sanofi), which have been approved by the European Union (EU), the US Food and Drug Administration (FDA) and the National Medical Products Administration of China (NMPA). Agalsidase α and agalsidase β are genetically homologous, similar in structure and function, and have the same amino acid sequence as the natural human α-Gal A.

Due to the low incidence of rare diseases, there are few clinical reports [22–24] on ERT for Fabry disease. This study aimed to explore the effectiveness and safety of ERT in clinical practice by comparing the involvement of organs such as the kidney, cardiac, and nervous systems before and after ERT and analyzing the incidence of ADR.

## Research object and method

### Object

Patients were diagnosed with Fabry disease based on the inpatient and outpatient treatment records of the Second Hospital of Hebei Medical University between March 2022 and November 2023. The inclusion criteria were: (1) clinical diagnosis of Fabry disease and (2) treatment, including ERT. Exclusion criteria included: (1) missing or incomplete clinical data caused by various factors, (2) clinical examination information less than 2 times, inability to compare the effectiveness before and after treatment, and (3) congenital diseases and other major diseases unrelated to Fabry disease.

This retrospective case study was approved by the Research Ethics Committee of the Second Hospital of Hebei Medical University (No.2023-R426), and the ethics committee approved that all participants were exempt from signing informed consent forms.

### Method

#### Data collection

The basic information, clinical data, laboratory examination data, imaging information, histopathological information, special examinations of Fabry disease (α-Gal A activity, GL-3/Lyso-GL-3 level, GLA gene detection, etc.), and AE and ADR were collected through the electronic medical record system. Data were statistically analyzed before and after ERT.

#### Diagnostic criteria

According to the “Chinese Expert Consensus on the Diagnosis and Treatment of Fabry disease [9], the diagnosis was confirmed by combining family genetic history, clinical manifestations, enzyme activity, biomarker levels, histopathological biopsy, and genetic testing. Diagnostic criteria included: decreased α-Gal A enzyme activity (which may be normal in 60% of female patients [25]), increased plasma GL-3/Lyso-GL-3 level (which may be normal in female patients [26]), osmiophilic “myeloid bodies” in the cytoplasm of histiocytes under an electron microscope, detection of pathogenic variants in the GLA gene, accompanied with or without relevant clinical symptoms of Fabry disease, such as peripheral neuropathic pain, hypohidrosis or anhidrosis, cutaneous angioceratoma, corneal turbidiform opacities, hearing loss, renal involvement, and cardiac hypertrophy. Genetic testing for pathogenic GLA mutations is an important diagnostic tool.

#### Determination of ERT effectiveness

Effectiveness evaluation indicators for Fabry disease include renal, cardiac, and nervous system evaluation indicators [27, 28].

##### Renal function evaluation indicators

The degree and progression of Fabry disease-related renal injury were monitored using the estimated glomerular filtration rate (eGFR) [29]. Based on eGFR, chronic kidney disease (CKD) can be divided into five stages [30, 31]: Stage CKD1 (≥ 90 mL/min/1.73 m^2^), CKD2 (60–89 mL/min/1.73 m^2^), CKD3 (30–59 mL/min/1.73 m^2^), CKD4 (15–29 mL/min/1.73 m^2^), and CKD5 (< 15 mL/min/1.73 m^2^). eGFR was calculated using the CKD-EPI formula. Other renal assessment indicators included [32]: microalbumin (mALB), 24 h urinary protein quantitation (24hPRO), urinary albumin/creatinine ratio (ACR), uric acid (UA), and β2 microglobulin (β2MG).

##### Cardiac evaluation indicators

Echocardiography has been used to evaluate the cardiac structure and ventricular function in patients with Fabry disease [33]. Cardiac structure assessment indicators included the left atrial diameter (LA) and left ventricular mass index (LVMI), where LVMI was calculated using the ventricular mass (LVM) correction of Deiereux and body surface area (BSA). The criteria for diagnosis of LVH based on LVMI [34] were ≥ 115 g/m^2^ for males and ≥ 95 g/m^2^ for females. Ventricular function was assessed by left ventricular ejection fraction (LVEF), e’ velocity of the interventricular septum, mean E/e’ and maximum tricuspid regurgitation velocity.

Laboratory data, including N-terminal B-BNP precursor (NT-proBNP) and serum troponin I (cTnI)/high-sensitivity troponin I (hs-cTnI) levels, were used to evaluate the degree and progression of Fabry cardiomyopathy/heart failure. The myocardial enzyme series included hypersensitive C-reactive protein (hs-CRP), serum myoglobin (MYO), creatine kinase (CK), serum creatine kinase isoenzyme (CK-MB), serum lactate dehydrogenase (LDH), and serum alpha-hydroxybutyrate dehydrogenase (HBDH), which were used to evaluate the degree and progression of myocardial injury and monitor therapeutic effects.

##### Neurological evaluation indicators

Pain improvement was evaluated according to the changes in clinical symptoms before and after ERT.

#### Safety analysis

According to ICH’s definition and terminology related to clinical trial safety [35], ERT safety was analyzed by collecting the incidence of AE, ADR, and serious adverse events or drug reactions (ASE) after ERT through an electronic medical record system. The decision to discontinue treatment, as well as subsequent management of AEs (including switching to other treatments) was based on the initial decision of the clinical physician.

### Statistical methods

SPSS21.0 software was used for the statistical analysis, and GraphPad Prism 8.0 software was used for drawing. Count data were expressed as the number of cases or percentages. If the differences in the data before and after treatment were normally distributed, a paired-sample t-test was used. If a normal distribution was not observed, the Wilcoxon rank-sum test was used. Statistical significance was set at *P* < 0.05.

## Results

### Clinical data

Twenty patients were clinically diagnosed with Fabry disease; four cases were excluded according to the inclusion and exclusion criteria, and 16 cases were included in this study. Basic information and clinical symptoms are shown in Table 1, and drug treatments and the prevention of ADR are shown in Table 2.

**Table 1:** Basic information and clinical symptoms of the patients

Subjects	Patients																Summary
	1	2	3	4	5	6	7	8	9	10	11	12	13	14	15	16	
Sex	M	M	M	F	M	M	M	M	M	M	M	M	M	F	F	F	
Age at ERT	54	51	34	41	48	11	12	10	15	39	42	20	20	59	57	33	
Family history	+	+	+	+	−	−	+ −	+	+	+ −	+	−	+	+	−	−	
Diagnostic indicators																	
α-Gal A/μmol/L/h	0.25	0.31	0.55			0.44	< 2.40	0.39	1	0.35	0.29	0.3			< 2.40	0.84	
Lyso-GL-3/ng/mL	135.5	140.3	57			85.2		100.6	100.6	346.4	72.8	114.5					
GLA gene mutation	+	+	+	+		+	+	+	+	+	+	+	+	+	+	+	
Pathological biopsy					Renal												
Early clinical symptoms																	
Peripheral nerve pain	+	+	+		+	+	+	+	+	+	+	+	+	+		+	14
Hypohidrosis/anhidrosis	+	+	+		+		+			+	+	+	+	+			10
Angiokeratoma							+				+	+	+	+			5
corneal whorl opacity	+	+		+	+							+					5
Decreased vision				+	+											+	3
Decreased hearing	+	+	+		+							+			+	+	7
Gastrointestinal dysfunction			+			+	+									+	4
Respiratory dysfunction	+	+								+							3
Serious complications																	
Kidney damage/failure	+	+	+		+				+	+	+				+		8
Myocardial injury/heart failure	+	+	+	+	+					+		+		+	+		9
Cerebral white matter damage/stroke	+	+	+	+	+					+				+	+	+	9

*M* male, *F* female, *α-Gal A* α-galactosidase A (reference range 2.40 − 17.65 μmol/L/h), *Lyso-GL-3* globotriaosylsphingosine (reference range < 1.11 ng/ml)

**Table 2:** Drug treatment

Subjects	Patients																Summary
	1	2	3	4	5	6	7	8	9	10	11	12	13	14	15	16	
*ERT*																	
Drugs	α	α	α	α	α	α	α	α	α	α	α	α	α	α	β	α	
Times	36	35	22	7	6	5	12	16	17	19	9	4	4	4	3	2	12.6 ± 10.9
Duration/W	89	89	58	15	12	10	28	48	48	50	35	17	7	7	6	2	32.6 ± 28.6
*Symptomatic treatment*																	
Renal system																	
ACEI/ARB	+	+			+				+		+				+		6
Renal dialysis			+							+							2
Cardiac system																	
Diuretics		+															1
ACEI/ARB	+	+													+		3
β receptor antagonists					+												1
Nervous system																	
Anticonvulsants					+		+			+		+	+				5
Anti-inflammatory analgesics			+			+										+	3
Antiplatelet	+	+								+	+				+	+	6
*Prevention of ADR*																	
Antihistamines	+	+	+	+	+	+	+	+	+	+	+	+			+		13
Glucocorticoids						+	+		+	+	+	+			+	+	8

*α* Agalsidase α, *β* Agalsidase β, *ACEI* angiotensin-converting enzyme inhibitor, *ARB* Angiotensin II receptor blocker, *ADR* Adverse drug reactions

#### Basic information and clinical symptoms of patients

The ages of the 16 patients, including four minors, ranged from 10 to 59 years. Moreover, there were 12 males and four females. Nine patients (56.3%) had a family history, and two patients (12.5%) had a suspected family history. Mutations in GLA were observed in 15 cases, and characteristic myeloid bodies were observed under an electron microscope in one case. Fourteen patients presented with childhood or adolescent disease onset, while two patients had adult-onset. Clinical symptoms are diverse and include peripheral neuralgia, angioceratoma, and corneal whorl opacity. Severe complications primarily involve the kidneys, cardiac system, and nervous system.

#### Medication treatment

All 16 patients were treated with ERT, including 14 patients treated with a concentrated solution of agalsidase α (0.2 mg/kg, IV infusion) and two patients treated with agalsidase β (1.0 mg/kg for the first time, 0.3 mg/kg for maintenance, IV infusion). The duration of ERT ranged from 2 to 36 weeks, and the duration ranged from 2 to 89 weeks.

Symptomatic treatment included treatment for kidney, heart, and nervous system symptoms. Of these, 13 patients (81.3%) received symptomatic treatment for the involvement of each organ, and 14 patients (87.5%) received anti-allergic pretreatment with antihistamines and glucocorticoids.

### Analysis of ERT effectiveness

#### Evaluation of renal indexes

**Fig. 1:**
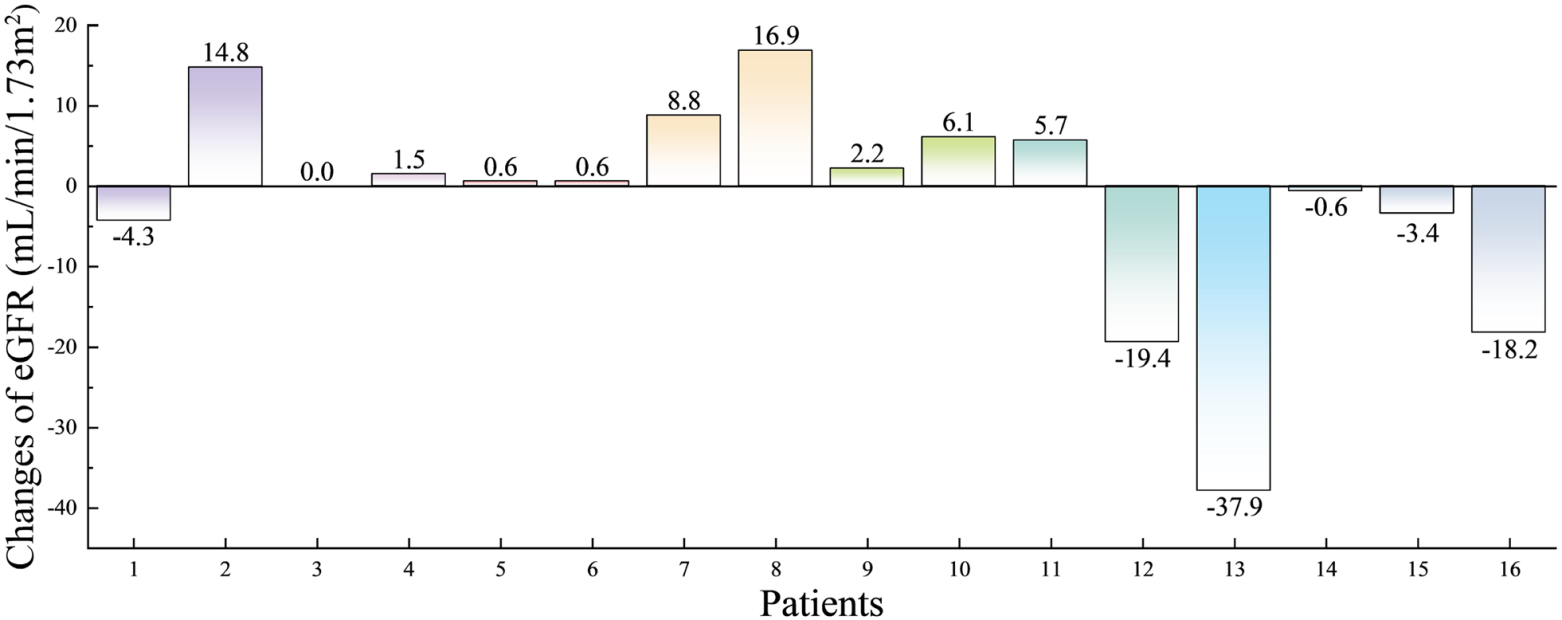
Changes in eGFR after ERT, *ERT* enzyme replacement therapy, *eGFR* estimated glomerular filtration rate

The baseline values of renal function and final data after ERT are shown in Table 3, and the changes in eGFR are shown in Fig. 1. No significant differences were observed in eGFR, mALB, 24hPRO, ACR, UA, and β2MG after ERT compared with the baseline values (*P* > 0.05). Renal function was stable or improved, and the CKD grade was improved in 2 cases and stable in 14 cases. Of the 11 patients who received 4 or more times of ERT, 10 (90.9%) had an increase in eGFR, and 4 of them were minors with an average increase of 7.12 mL/min/1.73 m^2^ after 22.5 weeks of treatment, with an annual eGFR increase of 16.5 mL/min/1.73 m^2^. The seven adult patients were treated for an average of 49.1 weeks, with an average eGFR increase of 3.5 mL/min/1.73 m^2^ and an annual eGFR increase of 3.7 mL/min/1.73 m^2^.

**Table 3:** Renal function data of patients at baseline and the last test during ERT

Subjects	Sort	Patients																Mean ± SD	*P*
		1	2	3	4	5	6	7	8	9	10	11	12	13	14	15	16		
eGFR(mL/min/1.73 m^2^)	Baseline	85.3	80.8	6.1	90.1	64.3	105.6	100.1	87.4	112.6	6.0	102.8	167.0	145.5	51.4	22.3	186.5	88.4 ± 51.8	0.836
	Last test	81.0	95.6	6.1	91.6	64.8	106.2	108.9	104.3	114.8	12.1	108.5	147.6	107.6	50.8	18.9	168.3	86.7 ± 46.1	
mALB(mg/L)	Baseline	395	139						1			664	9	24	7	967	23	248 ± 353	0.208
	Last test	166	209						1			1257	35	6	8	3072	30	531 ± 1033	
24 h PRO(g/24 h)	Baseline	1.38	0.51			1.49	0.10			0.44		1.16	0.09	0.24	0.10	3.72	0.36	0.87 ± 1.1	0.593
	Last test	1.29	1.30			0.69				0.44								0.93 ± 0.4	
ACR(mg/g)	Baseline	913	367						4			788	9	8	15	3393	25	614 ± 1102	0.859
	Last test	623	106						4			1990	11	4	9	4999	52	867 ± 1681	
UA(μmol/L)	Baseline	250	461	298	217	456	320	265	233	343	433	430	269	151	267	438	139	311 ± 106	0.918
	Last test	281	411	279	233	502	219	255	208	339	197	406	291	169	351	476	203	301 ± 103	
β2MG(mg/L)	Baseline	2.7	1.6	43.6	1.8	3.5	1.5	1.9	2.3	2.5	24.2	3.8	1.4	1.2	2.3	6.6	1.4	6.4 ± 11.4	0.732
	Last test	2.6	2.4	48.7	1.5	3.4	1.8	1.3	1.5	1.6	47.4	3.1	1.4	1.1	1.9	6.7	1.5	8.0 ± 15.7	

*ERT* enzyme replacement therapy, *eGFR* estimated glomerular filtration rate, *mALB* microalbumin (reference value 0.0–30.0 mg/L), *24 h PRO* 24 h urine protein quantification (reference value 0.00–0.15 g/24 h), *ACR* urinary albumin/creatinine ratio (reference value 0.0–30.0 mg/g), *UA* uric acid (reference value 208–428 μmol/L), *β2 mg* β2 microglobulin (reference value 0.8–2.8 mg/L)

#### Evaluation of cardiac indexes

**Fig. 2:**
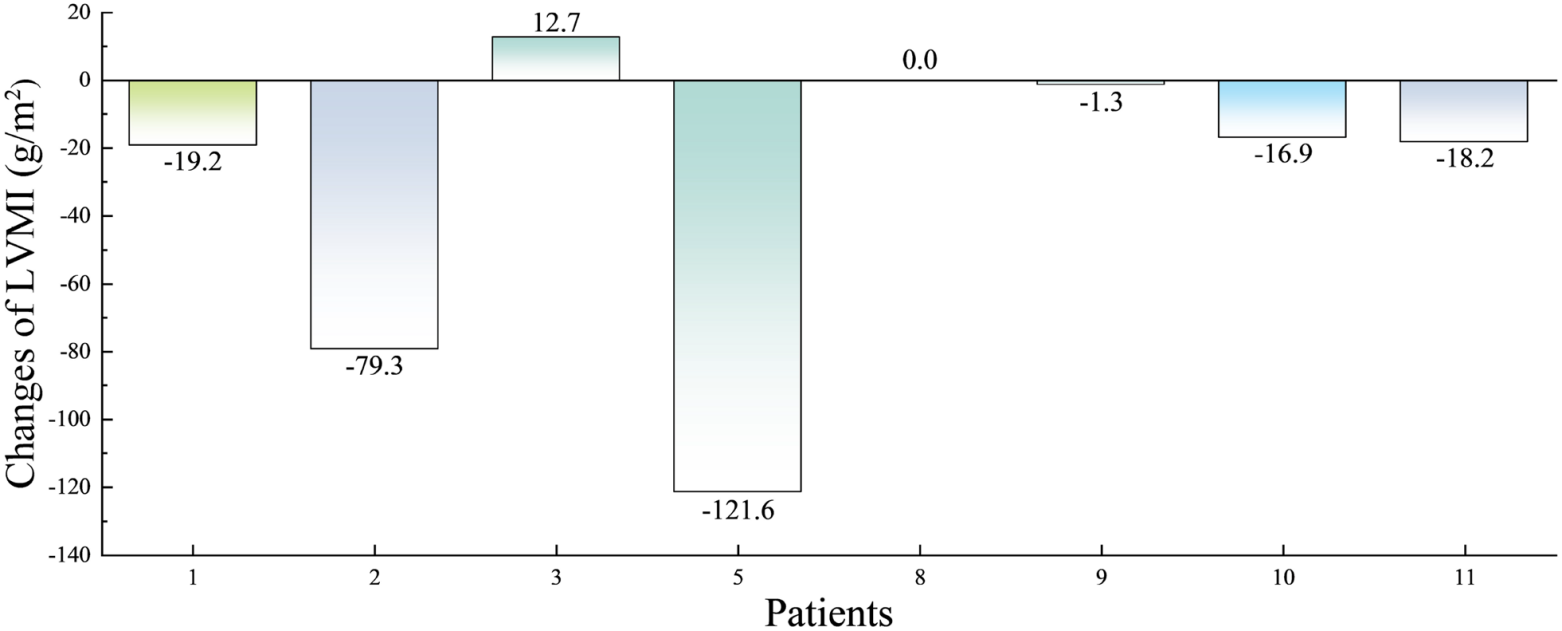
Changes in LVMI after ERT, *ERT* enzyme replacement therapy, *LVMI* left ventricular mass index

The echocardiographic results of eight patients were available, and their baseline values and data after the last treatment are represented in Table 4. Compared to the baseline value, LVMI decreased significantly after ERT (*P* = 0.043), and the change in LVMI is shown in Fig. 2. Among them, four patients with LVH (No. 1, 2, 5, 9) had a significant decrease in LVMI, while four patients without LVH fluctuated within the normal range. The LA and LVEF values of eight patients fluctuated within the normal range, and ERT maintained the interventricular septal e’ velocity, mean E/e’, and maximum tricuspid regurgitation velocity relatively stable.

**Table 4:** Cardiac function data of patients at baseline and the last test during ERT

Subjects	Sort	Patients																Mean ± SD	*P*
		1	2	3	4	5	6	7	8	9	10	11	12	13	14	15	16		
*Echocardiographic data*																			
LWMI(g/m^2^)	Baseline	240	298	97	116	191	69	67	87	162	98	108	148	258	71			144 ± 76	0.043
	Last test	221	218	110		70		67	86	146	79							125 ± 64	
LA(mm)	Baseline	39	39	31	30	47	29	29	35	28	34	32	35	45	33			35 ± 6	0.924
	Last test	50	39	38		34		28	31	30	34							36 ± 7	
LVEF(%)	Baseline	62.0	61.0	67.5	66.2	55.7	61.0	64.0	60.8	59.1	68.9	69.8	71.8	75.3	61.3			64.6 ± 5.5	0.597
	Last test	66.7	67.5	56.3		71.0		67.1	61.5	58.9	62.6							64.0 ± 5.0	
e’(cm/s)	Baseline	6.0	6.0	8.0	8.0	6.5	15.5	15.0	12.0	6.0	6.0	10.0	8.0	6.0	7.0			8.6 ± 3.3	0.677
	Last test	5.0	4.5	8.0		7.0		11.0	15.0	5.5	7.0							7.9 ± 3.5	
E/e’	Baseline	12.3	13.0	9.4	9.5	17.1	7.3	7.0	7.6	7.6	13.8	9.4	9.8	9.5	12.8			10.4 ± 3.0	0.825
	Last test	15.0	20.9	8.6		11.0		9.0	6.1	9.8	10.2							11.3 ± 4.6	
TR Vmax(cm/s)	Baseline	256	267				210	217	236	259		290						248 ± 29	0.590
	Last test	262	238					227	230	258								243 ± 16	
*Cardiac function laboratory data*																			
cTnl(ng/mL)	Baseline		0.15	0.03			< 0.01	< 0.01	< 0.01	< 0.01	0.05	0.03	0.01	< 0.01	0.02	0.26	< 0.01	0.1 ± 0.1	–
	Last test		0.15	0.02			< 0.01	< 0.01	< 0.01	< 0.01	0.03	0.02	< 0.01	< 0.01	0.03	0.25	< 0.01	0.1 ± 0.1	
hs-cTnl(ng/L)	Baseline	120			4.5	216												113 ± 106	–
	Last test	121			5.2	340												155 ± 170	
NT-proBNP(pg/mL)	Baseline	501	733	525	77	860	< 10	< 10	< 10	< 10		77	34	20	321	3842	56	640 ± 1103	0.670
	Last test	529	522	318	107	893	< 10	< 10	< 10	< 10		50	20	11	174	4000	236	624 ± 1152	
hs-CRP(mg/L)	Baseline	24.9	0.7	6.3	2.0	2.9	1.3	0.1	0.1	1.8	1.3	2.3	0.5	0.7	12.3	1.0	13.1	4.5 ± 6.8	0.959
	Last test	6.9	1.3	3.4	1.5	1.1	1.5	0.6	0.3	0.3	6.4	2.9	4.0	0.3	0.3	1.5	17.1	3.1 ± 4.3	
MYO(ng/mL)	Baseline	103	105	500	51	129	17	32	14	21	441	107	28	43	41	124	26	111 ± 146	0.214
	Last test	93	91	459	28	91	19	28	23	35	704	65	27	41	47	118	24	118 ± 189	
CK(U/L)	Baseline	103	139	66	59	77	79	81	30	50	120	125	105	63	81	71	24	80 ± 33	0.208
	Last test	111	175	75	76	89	86	78	51	64	106	74	86	84	131	84	24	87 ± 34	
CK-MB(U/L)	Baseline	7	20	23	10	22	6	14	10	17	18	11	18	15	14	11	16	15 ± 5	0.523
	Last test	16	24	14	12	17	8	15	15	10	13	18	12	12	4	10	17	14 ± 5	
LDH(U/L)	Baseline	316	266	222	201	235	174	166	181	281	207	179	191	148	223	259	165	213 ± 48	0.031
	Last test	275	273	190	203	250	170	145	165	174	146	179	126	140	245	263	146	193 ± 52	
HBDH(U/L)	Baseline	199	226	176	153	186	113	115	122	185	164	144	142	106	177	218	98	158 ± 40	0.510
	Last test	240	234	155	155	164	106	109	142	142	126	144	132	116	203	201	95	154 ± 44	

*ERT* enzyme replacement therapy, *LVMI* left ventricular mass index, *LA* left atrial diameter (reference value 19–35 mm), *LVEF* left ventricular ejection fraction (reference value 50.0–75.0%), *e′* ventricular septal velocity (reference value < 7.0 cm/s), *E/e′* reference value > 14.0, *TR Vmax* Tricuspid regurgitation velocity (reference value > 280 cm/s), *cTnl* serum troponin I quantification (reference value 0.00–0.04 ng/mL), *hs-cTnl* high-sensitivity troponin I (reference value 0.0–53.5 ng/L), *NT-proBNP* BNP-precursor brain natriuretic peptide (reference value 0–103 pg/mL), *hs-CRP* high-sensitivity C-reactive protein (reference value 0.0–6.0 mg/L), *MYO* myoglobin (reference value 0–85 ng/mL), *CK* creatine kinase (reference value 50–310 U/L), *CK-MB* creatinine kinase-MB subtype (reference value 0–24 U/L), *LDH* lactate dehydrogenase (reference value 120–250 U/L), *HBDH* α-hydroxybutyrate dehydrogenase (reference value 72–182 U/L)

The data from the laboratory tests of cardiac function are shown in Table 4. Compared with the baseline value, LDH decreased significantly after ERT (*P* = 0.031), and other indicators showed an improvement trend or remained stable; however, the difference was not statistically significant (*P* > 0.05).

#### Evaluation of the nervous system

Of the 16 patients, 14 developed peripheral nerve pain, most of which worsened during adolescence and decreased or disappeared during adulthood. Only four minor patient responses on the degree of limb pain were collected, of which three cases were slightly better than before and one case had no obvious change.

### Safety evaluation

None of the 16 patients experienced AE during ERT, and 14 were pretreated with antihistamines and glucocorticoids to prevent infusion-related AE.

## Discussion

ERT is a major method for treating Fabry disease, and the therapeutic drugs glycoside α and glycosidase β were approved by the NMPA of China in 2020 and 2019, respectively. Due to the short launch time, ERT in China has limited clinical experience. Although relevant studies have been reported in the United States and Europe, it is necessary to conduct clinical studies on the effectiveness and safety of ERT in Asian populations, considering racial differences.

### Treatment of Fabry disease

The treatment goal for Fabry disease is to improve clinical symptoms, delay the disease process, reduce organ damage, and improve quality and length of life. The treatment strategy is a combination of symptomatic treatment and specific treatment [35–37]. Symptomatic treatments mainly involve the involvement of various organs to provide analgesia, protein reduction, heart failure prevention, stroke prevention, and other symptomatic treatments. Specific therapies include ERT [38], drug molecular chaperone therapy [39, 40], substrate reduction therapy, and gene therapy [41, 42], which can alleviate organ damage by increasing or restoring α-Gal A enzyme activity and reducing substrate GL-3 storage, bringing new hope to patients with Fabry disease.

### Analysis of ERT effectiveness

#### Renal benefit

The accumulation of GL-3 in renal cells of patients with Fabry disease can cause proteinuria, interstitial fibrosis, glomerulosclerosis, and renal tubular atrophy, leading to the deterioration of renal function [43], which is characterized by a decrease in eGFR of more than 3 mL/min/1.73 m^2^ per year. The goal of treatment is to stabilize the eGFR and delay the progression of CKD. In this study, the CKD grade and renal function indices remained stable in all 16 patients who underwent ERT. We observed a large decrease in eGFR in 4 patients after prolonged suspension of ERT and an increase in eGFR in 2 of these patients after restarting ERT, suggesting that ERT is effective in improving renal function. In this study, the average annual eGFR increase of underage patients (< 18 years old) was more than that of adult patients, suggesting that ERT has improved benefits on the renal function of underage patients, which is consistent with the literature [44–46]. The accumulation of GL-3 and lyso-GL-3 in renal cells leads to irreversible kidney damage, and early ERT can inhibit or delay kidney damage [47]. Therefore, early diagnosis and ERT are extremely important to delay the progression of Fabry disease.

#### Cardiac benefit

Cardiac involvement in Fabry disease is an important cause of death and shortens life expectancy [48]. The accumulation of the substrates GL-3 and Lyso-GL-3 affects cardiomyocytes, leading to a series of diseases, such as left ventricular hypertrophy, conduction disorders, valve regurgitation, and heart failure. Cardiac symptoms occur in 40–60% of patients, usually in adulthood, around 30–40 years for men and 10 years later for women [49]. In this study, nine patients had cardiac involvement, with a mean age of 41 years in men and 52 years in women.

Cardiomyopathy in Fabry disease is characterized by increased LVH and LVM. Approximately one-third of female patients and half of male patients develop LVH-related cardiac symptoms [50]. The goal of treatment is to normalize LVH or prevent its progression, and reduce the incidence of cardiovascular events and the risk of cardiovascular disease recurrence [50, 51]. In this study, LVMI and LDH decreased significantly after ERT, among which LVMI decreased significantly in 4 patients with LVH, LVMI remained stable in four patients without LVH, and the cardiac benefit of patients was better than that reported in the literature [52, 53]. We observed a decrease in LVMI in one patient treated with continuous ERT for 30 weeks and an increase in LVMI after 18 weeks of treatment interruption, suggesting that ERT is effective in improving LVMI.

#### Improvement of pain and life quality

ERT has a significant effect on reducing neuropathic pain and can significantly reduce dependence on neuropathic pain drugs [54]. This study observed that a few adult patients experienced limb pain during childhood, which worsened during adolescence and improved during adulthood. A possible reason is that early pain in patients with Fabry disease is due to peripheral sensitivity, and a few small nerve fiber injuries lead to abnormal excitation and pain; however, with age and disease progression, extensive small nerve fiber damage leads to complete loss of function, and pain may be reduced [24].

### Safety of ERT

Due to the immunogenicity of ERT therapeutic drugs, anaphylactic infusion reactions can easily occur during ERT treatment, including chills, flushing, fever, nausea, dizziness, sweating, and fatigue. Oral or intravenous administration of antihistamines and glucocorticoids for pretreatment, reducing the infusion speed, and prolonging the infusion time of ERT can prevent ADR^0^. Fourteen patients were administered antihistamines or glucocorticoids. No AE and ADR were observed at the end of the study, indicating that ERT was safe.

### Limitations

This was a single-center study with certain limitations due to the minimal incidence of rare diseases, the small sample size, and the lack of analysis of cardiovascular risk factors, renal risk factors, and drug combinations. This was a retrospective cohort study, and the data on plasma levels of the biomarker GL-3/Lyso-GL-3 before and after ERT, heart failure classification, angina score, pain assessment scale, and quality of life score were incomplete; therefore, the results should be interpreted with great caution. These factors may limit the generalizability of the study’s results, and the clinical benefits of ERT need to be confirmed in larger multicenter clinical trials.

## Conclusions

ERT is an effective method to delay the progression of Fabry disease. Early diagnosis and initiation of ERT are important for stabilizing or improving renal function in patients with Fabry disease. ERT can significantly reduce LVMI and LDH levels and improve heart function in patients with Fabry disease. ERT can relieve neuropathic pain and improve the patient’s quality of life. No AE and ADR were observed from the beginning of ERT to the end of the study. The clinical effectiveness and safety of ERT have been previously demonstrated. This study supplements the efficacy and safety data of ERT in the Asian population, which can provide reference for the drug treatment of Fabre’s disease in Asians. This study has certain practical significance and novelty.

## Data Availability

All raw data for this study can be obtained at the following link: https://datadryad.org/stash/share/qBws_7lbta_QoaH-ChUcwF8e5xBuiKhT0okI8Pt628E.
